# Endothelial glycocalyx shields the interaction of SARS-CoV-2 spike protein with ACE2 receptors

**DOI:** 10.1038/s41598-021-91231-1

**Published:** 2021-06-09

**Authors:** Marta Targosz-Korecka, Agata Kubisiak, Damian Kloska, Aleksandra Kopacz, Anna Grochot-Przeczek, Marek Szymonski

**Affiliations:** 1 Department of Physics of Nanostructures and Nanotechnology, Faculty of Physics, Astronomy and Applied Computer Science, Jagiellonian University, Kraków, Poland; 2 Department of Medical Biotechnology, Faculty of Biochemistry, Biophysics and Biotechnology, Jagiellonian University, Kraków, Poland

**Keywords:** Nanoscale biophysics, Single-molecule biophysics, Biophysics, Glycobiology, Mechanisms of disease, Atomic force microscopy

## Abstract

Endothelial cells (ECs) play a crucial role in the development and propagation of the severe COVID-19 stage as well as multiorgan dysfunction. It remains, however, controversial whether COVID-19-induced endothelial injury is caused directly by the infection of ECs with SARS-CoV-2 or via indirect mechanisms. One of the major concerns is raised by the contradictory data supporting or denying the presence of ACE2, the SARS-CoV-2 binding receptor, on the EC surface. Here, we show that primary human pulmonary artery ECs possess ACE2 capable of interaction with the viral Spike protein (S-protein) and demonstrate the crucial role of the endothelial glycocalyx in the regulation of the S-protein binding to ACE2 on ECs. Using force spectroscopy method, we directly measured ACE2- and glycocalyx-dependent adhesive forces between S-protein and ECs and characterized the nanomechanical parameters of the cells exposed to S-protein. We revealed that the intact glycocalyx strongly binds S-protein but screens its interaction with ACE2. Reduction of glycocalyx layer exposes ACE2 receptors and promotes their interaction with S-protein. These results indicate that the susceptibility of ECs to COVID-19 infection may depend on the glycocalyx condition.

## Introduction

The Severe Acute Respiratory Syndrome Coronavirus 2 (SARS-CoV-2) enters the host cell using the angiotensin-converting enzyme 2 (ACE2) receptors^1–3^. The virus attaches to cells via spike proteins (S-proteins). Structurally, S-proteins are glycoproteins that protrude from the viral surface and form a characteristic crown. S-protein consists of two subunits: S1 subunit that includes receptor-binding domain (RBD) and that recognizes and binds to the specific receptor; S2 subunit that regulates the membrane fusion between the virus and host cells^4^. The interaction between S-protein and ACE2 has a pivotal role in virus pathogenesis. Therefore S-protein has been considered as a promising therapeutic target for the development of vaccines and antiviral compounds. Moreover, the high transmissibility of SARS-CoV-2 and the severity of COVID-19 is related to the high binding affinity of S-protein to ACE2 receptors^5^.

SARS-CoV-2 belongs to respiratory viruses, which are transmitted mainly through respiratory droplets. Therefore, airway epithelial cells are the main target that is initially exposed to contact with viruses and are responsible for the initiation of COVID-19 disease and development of severe acute respiratory syndrome^6–10^. While the role of the airway epithelium is essential in the initial COVID-19 stage, the endothelium may play a crucial role in the development and propagation of the later severe COVID-19 stage^5,11^.

The strong inflammatory response induced by SARS-CoV-2 viruses causes endothelial dysfunction and promotes severe cardiovascular complications such as venous thromboembolism, arrhythmias, and myocardial dysfunction, as evidenced by clinical observations^12–15^. However, the mechanism of SARS-CoV-2-induced damage of ECs remains an open question, hypothesizing direct infection of ECs by the virus and their subsequent injury or indirect mechanisms originating, for example, from immune processes^16^. This issue is fueled by contradictory data on the presence of ACE2 receptor on ECs^4,16–23^, and the potential viral infection of these cells^24–27^. Some studies indicate that ECs can be a direct target of SARS-CoV-2 infection. Post-mortem histological analysis evidenced a direct viral infection of ECs and diffuse endothelial inflammation^25,26^. Moreover, SARS-CoV-2 viremia was recently reported^28^ and patients with measurable levels of the SARS-CoV-2 RNA in serum were identified to be at a higher risk of progression to a critical disease and death^29^. However, the other studies suggest that ECs are resistant to infection with the SARS-CoV-2 virus^16,27^, and the vascular dysfunction seen in severe COVID-19 may be a result of circulating inflammatory mediators released by other infected cells^27^.

ECs are covered by the glycocalyx, a sugar-rich brush layer that tightly overlays the luminal surface of the endothelium^30^. The glycocalyx is composed mainly of glycoproteins and proteoglycans that form a protective shell surrounding the cell^31^. The glycocalyx is the first line of cellular defence against infection. However, some of the molecules building glycocalyx also play a role as receptors in intercellular interactions, including interaction with viruses^32–36^. Heparan sulfate (HS) is the component of glycocalyx contributing to the interaction with coronaviruses as a co-receptor in epithelial cells^37^. In the context of the SARS-CoV-2 infection, it was reported that HS proteoglycans promote the interaction of S-protein with ACE2 and are required for SARS-CoV-2 virus binding and infection of epithelial cells, alveolar macrophages and dendritic cells^28,29^. Additionally, heparin blocked SARS-CoV-2 infection of epithelial cells and alveolar macrophages^28^. However, the role of the glycocalyx in the binding of SARS-CoV-2 to endothelium and its relation to ACE2 binding is still unclear and understudied.

Adhesive interactions in biological systems can be monitored using the force spectroscopy method with an atomic force microscope (AFM). For many years, AFM based force-spectroscopy has been used to monitor the interactions between individual cells (single-cell force spectroscopy, SCFS)^40,41^ or to study the bonds between individual molecules (single-molecule force spectroscopy SMFS)^42^. Moreover, very recently, it was demonstrated that single-molecule AFM force spectroscopy could be used to investigate molecular interaction and inhibition of SARS-CoV-2 binding to the ACE2 receptor for A549 cells^43^.

In this work, by using the AFM force spectroscopy method, we directly measured the adhesive interactions between the S-protein of SARS-CoV-2 and the surface of ECs. In particular, we focused on the interaction with the ACE2 receptors and the role of the cellular glycocalyx either as a shield or as a potential co-receptor for the S-protein binding. For this purpose, we used a colloidal AFM probe decorated by S-protein as a sensor for both glycocalyx detection and monitoring the adhesive interaction of S-protein with the cell surface. The use of the same AFM probe enabled us to simultaneously perform cell elasticity analysis, which is a relatively novel biomarker of endothelial dysfunction.

## Results

**Figure 1:**
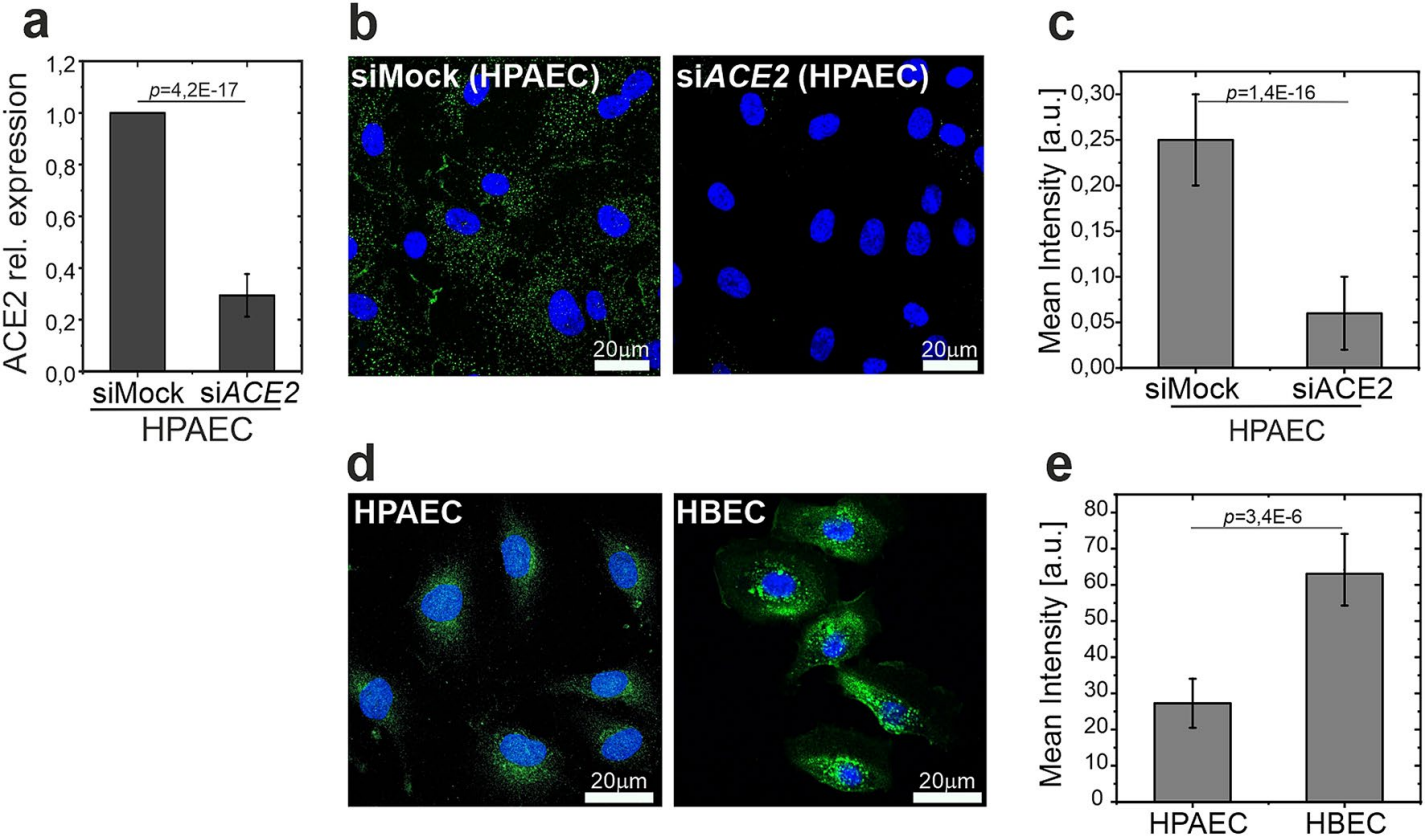
Examination of the presence of ACE2 in human ECs. (**a**) Expression of *ACE2* in siMock and si*ACE2-*transfected HPAECs. (**b**,**c**) Immunofluorescent staining of HPAECs transfected with ACE2-targeting (si*ACE2*) and non-targeting (siMock) siRNA, with anti-ACE2 antibody. Representative photographs and quantitative analysis of immunofluorescence intensity. (**d**,**e**) Immunofluorescent staining of ACE2 in HPAECs and HBECs. Representative photographs and quantitative analysis of immunofluorescence intensity. Statistics: *p* values were determined by one-way ANOVA followed by Tukey’s post-hoc test. Figure created with OriginPro2021 (https://www.originlab.com/2021) and ImageJ 1.53e (https://imagej.nih.gov/ij/).

To examine the presence of ACE2 in human ECs, primary human pulmonary artery ECs (HPAECs) transfected with non-targeting (siMock) or *ACE2*-targeting (si*ACE2*) siRNA (Fig. 1a) were stained with anti-ACE2 antibody (Fig. 1b,c). The staining confirmed the presence of ACE2 in HPAECs. Compared to human bronchial epithelial cells (HBECs), the level of ACE2 in HPAECs was significantly lower (Fig. 1d,e). Moreover, unlike in HBECs, the surface distribution of ACE2 in HPAECs was uniform (Fig. 1d).

**Figure 2:**
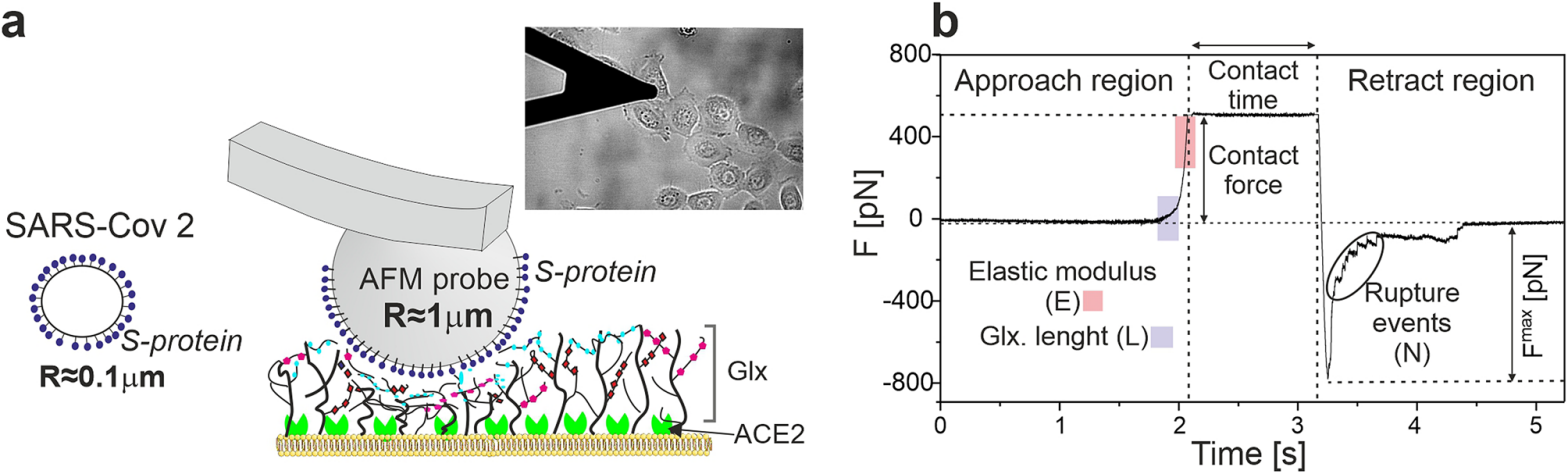
Experimental design and characterization of studied HBEC and HPAEC cells. (**a**) The idea of the experimental setup. Multiple spike proteins are attached to a large spherical AFM probe that is approached to the cell surface. Inset: Optical image of the experimental setup with a spherical probe. The AFM cantilever with a probe and HBEC cells on the glass are immersed in the HBSS solution. (**b**) Time diagram of recording a single force-distance curve with the approach, contact and retract regions. In the approach part, regions of curves taken for analysis of glycocalyx parameters (blue) and cell elasticity (red) are schematically marked. The maximal de-adhesive force and rupture events are marked on the retracting part of the curve. Figure created with OriginPro2021 (https://www.originlab.com/2021) and Corel Draw2020 (https://www.coreldraw.com/pl/).

To address the question of potential SARS-CoV-2 interaction with ECs, the adhesive interactions between the S-protein of SARS-CoV-2 and EC surface were measured. We designed a protocol that is illustrated in Fig. 2a. A spherical AFM probe with a radius of approximately 1 µm was covered with S-protein. This relatively large probe diameter, which is an order of magnitude larger than the size of SARS-CoV-2 virus, enables the enhancement of the adhesion signal. Simultaneously, the probe diameter is still small enough to obtain spatially resolved information at a length scale of a single EC. Moreover, using a spherical probe with a large diameter allowed us to sense and characterize the structural parameters of the soft glycocalyx layer for the same sample areas.

An exemplary experimental force curve (Fig. 2b) represents the time-dependence of the force that acts on the AFM probe during approach and retract processes. From the approach part of the curve, the effective elastic modulus of the cell (E) and the length (L) of glycocalyx are extracted using Hertz and Alexander-de Gennes models^44^, respectively. Adhesion parameters (F^max^—maximum detachment force, N—number of rupture events) are derived from the fragment of the curve measured in the retracting phase.

To verify the sensitivity of the setup to S-protein/ACE2 interaction, we performed measurements for HBECs, for which strong adhesive interactions can be expected^39^. Since the S-protein binding to ACE2 receptors plays a pivotal role in the SARS-CoV-2 entry to the host cell, we used the anti-ACE2 blocking antibody (the same one which was used for the immunofluorescent detection of ACE2, Fig. 1) to prevent the specific interaction of S-protein with ACE2 receptors.

**Figure 3:**
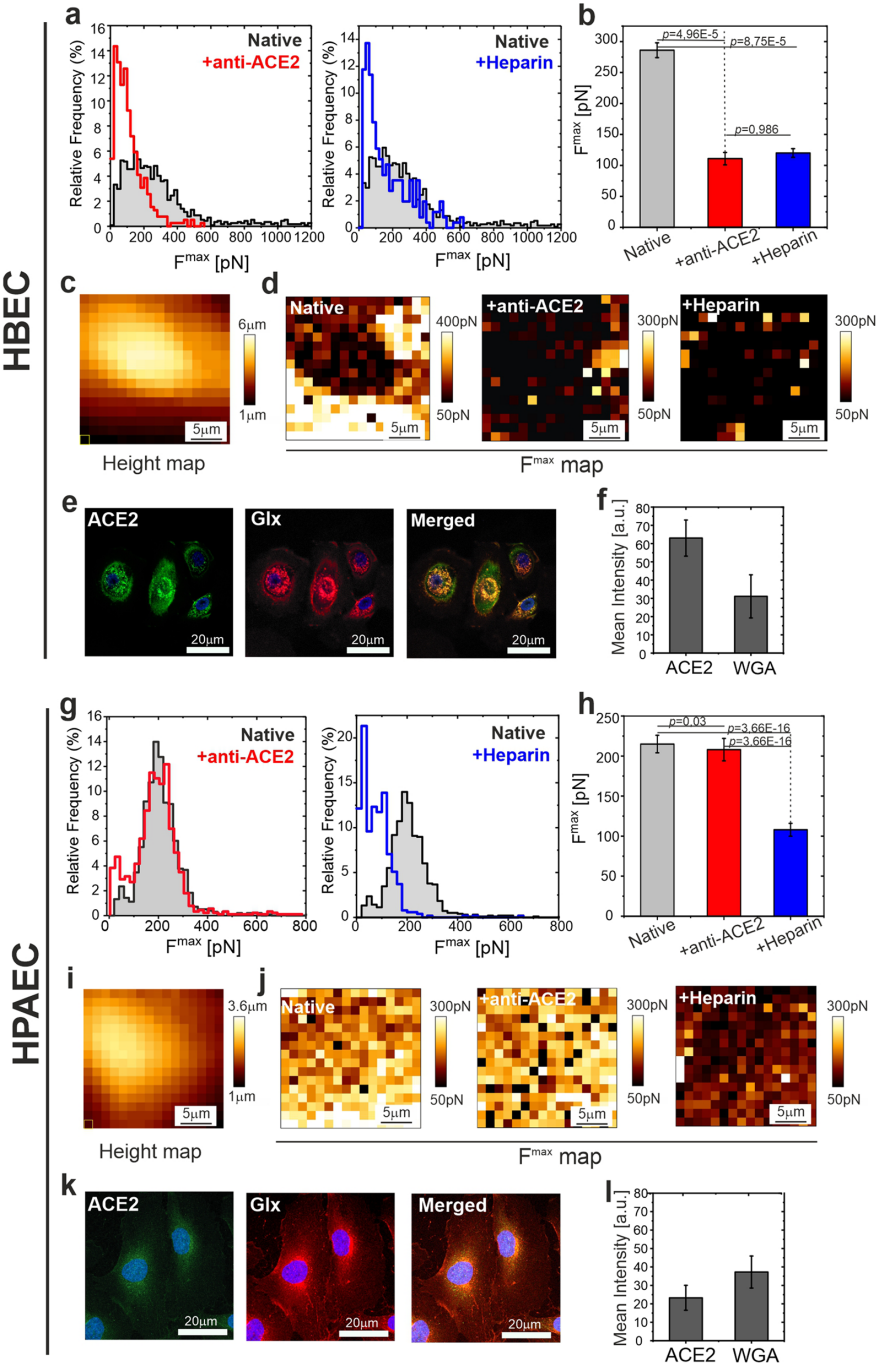
Differences in adhesive interactions between the S-proteins of SARS-CoV-2 and the surfaces of human bronchial epithelial cells (HBECs) and human pulmonary artery endothelial cells (HPAECs). (**a**) Histograms of the maximal detachment force F^max^ for HBECs. Left: comparison of data for native and anti-ACE2 treated cells. Right: comparison of data for native and heparin treated system. (**b**) Comparison of mean values determined from histograms. (**c**) AFM height map measured for a single HBEC cell. (**d**) Corresponding adhesive maps measured for this cell. Left: native cell. Middle: anti-ACE2 treatment. Right: heparin treatment. (**e**) Fluorescent staining of ACE2 (green, left column), glycocalyx (Glx, red, middle column) and Merged (right column). (**f**) Quantitative data of ACE2 and Glx mean fluorescence intensity. (**g**) Histograms of the maximal detachment force F^max^ for HPAECs. Left: comparison of data for native and anti-ACE2 treated cells. Right: comparison of data for native and heparin treated system. (**h**) Comparison of mean values determined from histograms. (**i**) AFM height map measured for a single HPAEC cell. (**j**) Corresponding adhesive maps measured for this cell. (**k**) Fluorescent staining of ACE2 (green, left column), Glx (red, middle column) and Merged (right column) (**l**) Quantitative data of ACE2 and Glx mean fluorescence intensity. Left: native cell. Middle: anti-ACE2 treatment. Right: heparin treatment. Statistics: *p* values were determined by one-way ANOVA followed by Tukey’s post-hoc test. Experimental details are listed in Supplementary Table 1. Source data are provided as a Source Data file. Figure created with OriginPro2021 (https://www.originlab.com/2021), ImageJ 1.53e (https://imagej.nih.gov/ij/) and JPK Data Processing 6.1.79 (https://www.jpk.com/).

A statistically significant decrease of the mean value of F^max^ denotes the sensitivity of the setup to S-protein/ACE2 interaction (for histograms of other parameters, maximal detachment force, rupture events, work of detachment, elastic modulus and glycocalyx length, see Supplementary Figure 1). Next, low molecular weight heparin (LMWH), which has a very similar structure to HS, was used as a non-specific blocker of S-protein^38^. Simultaneous preincubation of the probe and cells with heparin reduced the mean detachment force to the level similar to that observed after ACE2 blocking with the antibody (Fig. 3a,b, Supplementary Table 2). The height map (Fig. 3c) and adhesive maps shown in Fig. 3d indicate that for native cells, the adhesive events were mainly recorded around the central part of the cells, in the perinuclear area, which was determined from the height map. After the addition of ACE2 blocking antibody or heparin, in both systems, a significant reduction of adhesive events was directly visible on the adhesive maps (Fig. 3d). Interestingly, the distribution of ACE2 on HBEC surface was not uniform. High ACE2 density was observed close to the nucleus, but in this region ACE2 staining colocalizes with glycocalyx, which is not the case in the perinuclear area more distal from the nucleus (Fig. 3e,f). It shows, that ACE2 may remain uncovered by glycocalyx in some parts of epithelial cells.

Contrary to HBECs, incubation of HPAECs with ACE2 blocking antibody had no significant effect on the maximum detachment force (Fig. 3g,h). A decrease in the adhesion between S-protein and the cell surface was observed in response to the heparin pretreatment. (simultaneous pretreatment of the probe and cells) (Fig. 3g–j, Supplementary Figure 2, Supplementary Table 2). Importantly, the inhibition of S-protein interaction with ECs was not related to the potential heparin-induced increase in glycocalyx length, since the preincubation of the probe only, but not HPAECs only, with heparin, decreased the maximal detachment force (Supplementary Figure 3). Spatially resolved maps of adhesion parameters showed much weaker spatial dependence of the adhesion parameters, with a much less pronounced increase of the adhesion in the perinuclear area, relatively to the nuclear region (Fig. 3i,j). It corresponds with the uniform coverage of ACE2 and glycocalyx on the EC surface (Fig. 3k). Comparison of mean fluorescence intensity is shown in Fig. 3l.

**Figure 4:**
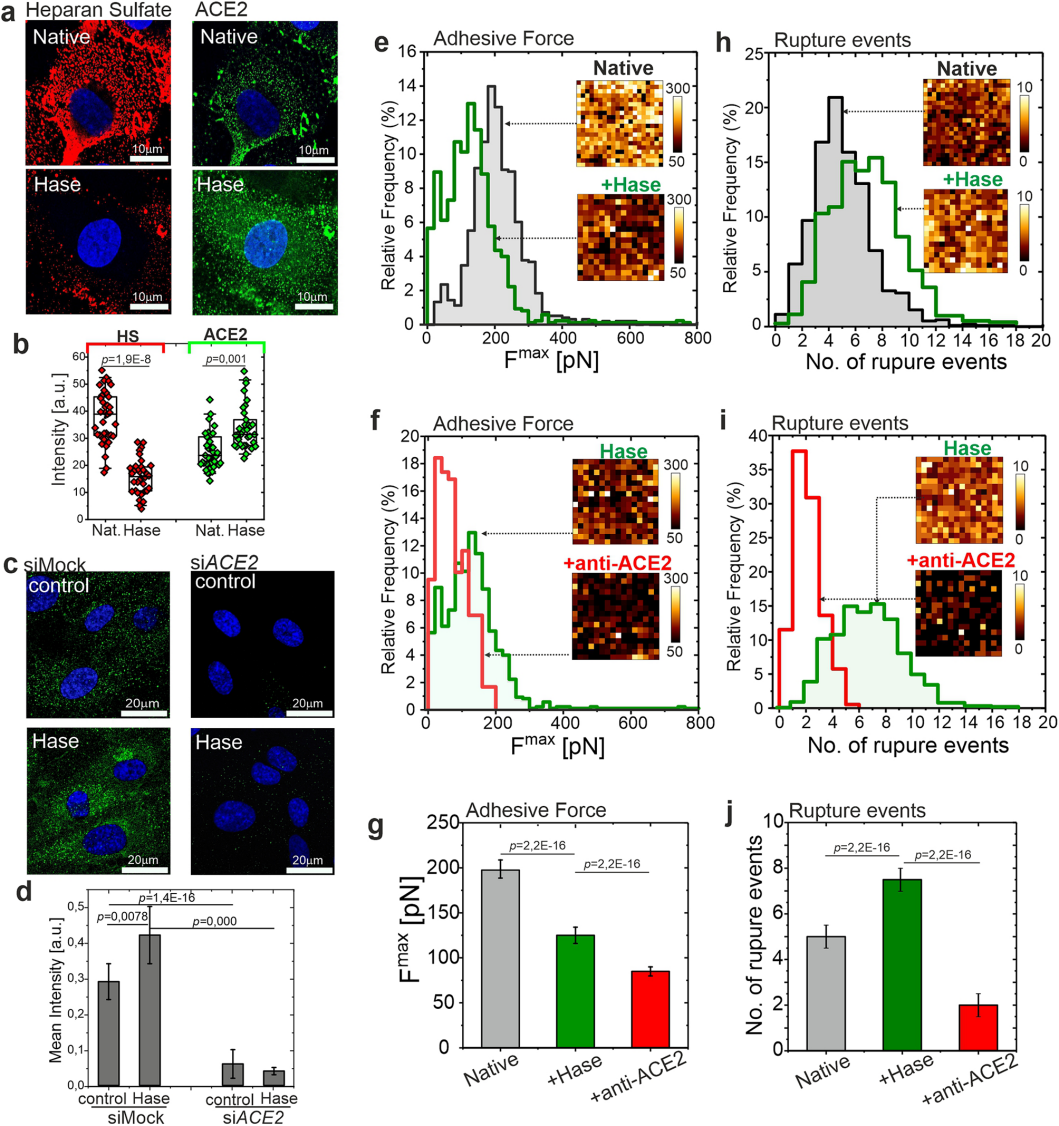
Removal of heparan sulfate from glycocalyx reduces the overall adhesion of S-protein to HPAECs but exposes ACE2 receptors for binding. (**a**) Fluorescence staining of HS (red, left column) and ACE2 receptors (green, right column) performed for native (top row) and heparinase (Hase) treated HPAECs (bottom row). (**b**) Quantitative data of HS and ACE2 mean fluorescence intensity. (**c**,**d**) Fluorescent staining of ACE2 in siMock and si*ACE2*-transfected HPAECs treated with heparinase. Representative pictures and quantitative data. (**e**,**h**) Histograms of adhesive parameters for native cells (grey) and heparinase treated cells (green). (**f**,**i**) Histograms of adhesive parameters for heparinase treated cells (green) and heparinase treated cells successively incubated with anti-ACE2. (**g**,**j**) Comparison of mean values. Left: plots for maximal detachment force. Right: plots for the number of rupture events. In all histograms and insets show selected spatially resolved maps (25 µm × 25 µm). Statistics: *p* values were determined by one-way ANOVA followed by Tukey’s post-hoc test. Experimental details are listed in Supplementary Table 1. Source data are provided as a Source Data file. *HS* heparan sulfate, *Hase* heparinase, *anti-ACE2* ACE2 blocking antibody, *Glx* glycocalyx. Figure created with OriginPro2021 (https://www.originlab.com/2021), ImageJ 1.53e (https://imagej.nih.gov/ij/) and JPK Data Processing 6.1.79 (https://www.jpk.com/).

To directly investigate whether a controlled degradation of the glycocalyx leads to a reduction of adhesive interactions, the enzyme heparinase was applied. Heparinase selectively sheds HS from the cell surface. Before AFM measurements, the reduction of HS by heparinase, and the ACE2 level was verified using fluorescence microscopy. Representative images of HS and ACE2 staining (Fig. 4a) and quantitative comparison (Fig. 4b) indicate a significant reduction of HS, confirmed by the AFM measurement of the glycocalyx length (L = (333 ± 3) nm vs (177 ± 8) nm, native vs heparinase, respectively), and an increase of ACE2 level on the HPAEC surface in response to heparinase treatment. The specificity of staining of heparinase-induced ACE2 exposure was confirmed with siRNA against *ACE2* (Fig. 4c,d).

For AFM measurements, the experiments with heparinase were carried out in two steps. At first, force-distance curves were collected for cells incubated with heparinase for selective glycocalyx removal. Subsequently, heparinase-preincubated cells were treated with anti-ACE2 blocking antibody to inhibit the specific interaction of S-protein with ACE2 receptors. Control experiments were done on native cells. The cumulative results obtained for all measured cells are shown in the form of histograms of maximum detachment force, and a number of rupture events (Fig. 4e–j). Insets in plots show selected spatially resolved maps (25 µm × 25 µm) of maximum adhesion force and a number of rupture events. Each histogram from the insets was constructed from multiple maps (Supplementary Table 1). Reduction of the glycocalyx layer by heparinase led to a decrease of the adhesive force (Fig. 4e–g) and a simultaneous increase in the number of rupture events (Fig. 4h–j). Contrary to cells with the intact glycocalyx, for cells pretreated with heparinase, ACE2 blocking caused a significant decrease in the adhesion force and the number of rupture events (Fig. 4e–j).

**Figure 5:**
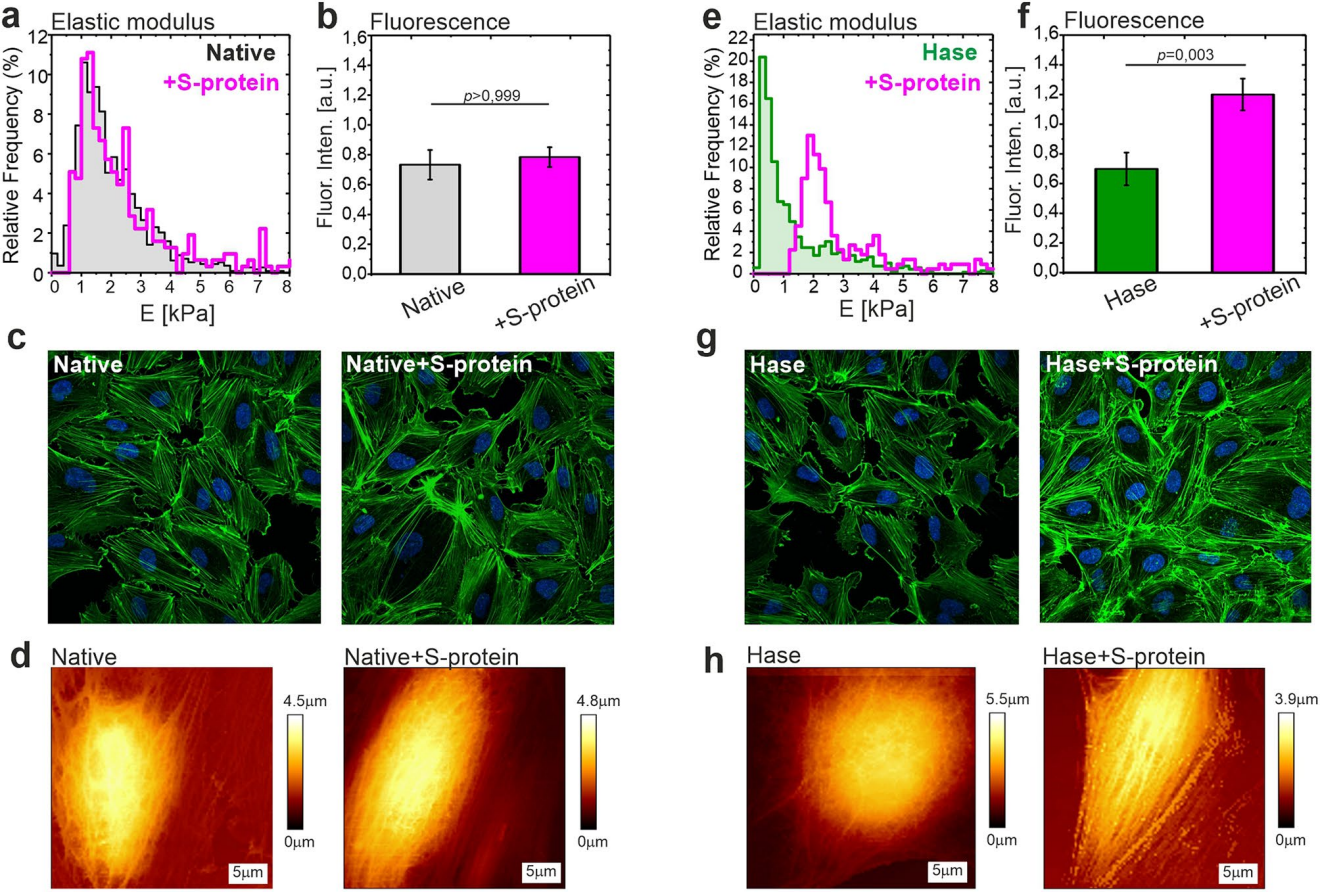
Endothelial cells stiffening after incubation with S-protein is more pronounced for cells with removed glycocalyx. (**a**) Elastic modulus of HPAECs for native cells (grey histogram) and for cells incubated with S-protein (magenta). (**b**) Mean fluorescence intensity of phalloidin (AlexaFluor488). (**c**) Examples of fluorescence images depict the actin structure in native HPAECs and after incubation with S-protein. Green—actin. Blue—nucleus. (**d**) Examples of AFM-QI images of native HPAECs and after incubation with S-protein. (**e**) Elastic modulus obtained for HPAECs pre-incubated with heparinase (green) and next incubated with S-protein (magenta). (**f**) Mean fluorescence intensity of phalloidin (AlexaFluor488) after removal of HS. (**g**) Fluorescence images show the actin polymerization that occurred after incubation with S-protein for HPAECs pre-incubated with heparinase. (**h**) Examples of AFM-QI images depict the changes of cell morphology and cortical actin network after incubation with S-protein for HPAECs pre-incubated with heparinase. Statistics: *p* values were determined by one-way ANOVA followed by Tukey’s post-hoc test. Figure created with OriginPro2021 (https://www.originlab.com/2021), ImageJ 1.53e (https://imagej.nih.gov/ij/) and JPK Data Processing 6.1.79 (https://www.jpk.com/).

Finally, the functional changes in the cells reflected the augmentation of the specific interactions of S-protein with ACE2 after the reduction of the glycocalyx. To verify our observations, we incubated HPAECs with S-protein and evaluated the cell elasticity with an uncovered AFM probe. Incubation of HPAECs with S-protein increased the cell membrane stiffness and actin polymerization only when the cells were pretreated with heparinase (Fig. 5).

## Discussion

In this paper, we confirmed the presence of ACE2 protein in ECs and revealed a significant role of the glycocalyx in the regulation of the binding of SARS-CoV-2 S-protein to the EC surface. In our experiments, multiple S-proteins were attached to a micron-sized probe, which enabled adhesive signal enhancement. Still, the AFM probe size was small enough to get spatially resolved information and directly sense the delicate glycocalyx layer. Therefore, this experimental design resembles typical setups for single-cell spectroscopy^40^ rather than single-molecule force spectroscopy^45–47^, which has been very recently applied to study the molecular interaction and inhibition of SARS-CoV-2 S-protein binding to the ACE2 receptor on A549 cells^43^.

We validated our experimental AFM setup on epithelial cells (HBECs). Results presented in Fig. 3a show the sensitivity of interaction between S-protein and ACE2 receptor, which is the principal mechanism responsible for the entry of the SARS-CoV-2 into airway epithelial cells. Blocking of the ACE2 receptor with anti-ACE2 antibody significantly reduced the total adhesion force. Spatially resolved adhesive maps collected for the native cells illustrate the accumulation of adhesive events in the perinuclear part of the cell, which may indicate receptor clustering. This agrees with the observed non-uniform spatial distribution of glycocalyx and high expression of ACE2 on HBECs. The receptor clustering increases the effectiveness of viral infection by raising the probability of virus binding and its entry to the cell. Blocking of S-protein with heparin decreased the total adhesion from (286 ± 12) pN to a value of (121 ± 6) pN. A similar effect was very recently reported for different types of epithelial cells^39^.

Our study revealed that the characteristics of the interaction of the S-protein with ACE2 differ between endothelial cells and epithelial cells. For ECs we observed uniform distribution of the glycocalyx layer, and low expression of ACE2 receptors. AFM measurements performed for HPAECs showed that blocking of ACE2 with anti-ACE2 does not change the S-protein binding to the EC surface. After blocking, a value of F^max^ = (190 ± 4) pN was observed, which was not statistically different from the value for native cells (F^max^ = (195 ± 2) pN). However, similarly to the HBECs, the addition of heparin blocked the interaction of S-protein with HPAEC surface, which denotes that for native ECs, the ACE2 protein had no measurable effect on S-protein adhesion. A similar effect was previously observed for HCoV-NL63 coronavirus^37^. Also, a very recent preprint, based on an in vitro study, suggested a resistance of native ECs to SARS-CoV-2, which is consistent with our observations^27^.

To explain this nonintuitive lack of the interaction of S-proteins with ACE2 for native ECs, we treated the cells with heparinase. Heparinase can selectively remove heparan sulphate, the main component of the endothelial glycocalyx. We hypothesized that the intact endothelial glycocalyx shields the ACE2 receptors. The glycocalyx is a dense, negatively charged brush surrounding the cell and forms a protective shield that may block the adhesive interaction with receptors located underneath. HS component of the glycocalyx has a global negative charge and therefore, can interact electrostatically with the viral S-proteins by binding with the positively charged domain of S-protein^39^. Simultaneously, for native “healthy” ECs the glycocalyx is long enough (L = (333 ± 3) nm according to Supplementary Figure 3 and Supplementary Table 2) to block the direct contact of S-protein and ACE2 membrane receptors that are directly anchored to the cell surface. Removal of HS from the endothelial glycocalyx by heparinase treatment confirmed our hypothesis. First, the removal led to a significant reduction in the total adhesion force. At the same time, the number of rupture events raised, which denotes an increase in specific interactions, likely with the ACE2 receptor. This is confirmed by the fluorescent staining of HS and ACE2, where also an effect of the glycocalyx reduction and an increased expression of ACE2 receptors can be observed. Most importantly, for cells pre-incubated with heparinase and with a partially removed glycocalyx, a successive blocking of ACE2 with anti-ACE2 resulted in a significant decrease of the maximum detachment force. This means that for the ECs with impaired glycocalyx, the interaction of S-protein and ACE2 is considerable.

Moreover, the results from the elasticity measurements (Fig. 5) indicate that the response of HPAECs to incubation with S-protein depends on the glycocalyx condition. Removal of HS from the glycocalyx layer strongly increased HPAEC stiffness, which correlated with the actin polymerization. EC stiffening is one of the symptoms of endothelial dysfunction, as it was shown in our previous papers^48,49^. The stiffening of HPAECs after incubation with S-protein can be indirectly linked with an onset of the endocytosis process, which takes place during SARS-CoV-2 entry into the cell. The binding of a specific ligand to the ACE2 receptors was shown to trigger internalization of the receptor^50,51^. In accordance, it was demonstrated that S-protein activates the ACE2 mediated cellular endocytosis signal pathway, by which SARS-Co-V enters the susceptible cells^52^. The endocytosis process induces the actin cytoskeleton polymerization and engages the actin fibers in multiple steps of internalization, endosomal sorting, and trafficking of viral particles in ECs^50^. In our experiments, for native ECs with a well-preserved glycocalyx layer, the addition of S-protein did not change the elastic modulus. Evaluation of the cell elasticity strengthened the hypothesis, that glycocalyx, by the strong binding of S-protein to HS, can prevent the interaction of S-protein with ACE2 receptors in ECs.

The influence of glycocalyx on the interaction between S-protein and ACE2 is of particular importance. Due to its location, the glycocalyx is the first target of the virus interaction, and therefore this interaction could determine the virus entry to the host cell. In this work, we have shown a dual role of the endothelial glycocalyx in control of the virus adhesion and interaction with ACE2 receptors. For healthy endothelium, the well-preserved glycocalyx layer promotes the overall adhesion of S-protein to the cell surface and acts as an “anchor”^53^. However, at the same time the rich endothelial glycocalyx screens the S-proteins from the ACE2 receptors. For well-preserved glycocalyx, ACE2 expression is low and glycocalyx acts as a structural barrier. For impaired glycocalyx, though the total adhesion of S-protein to the cell surface is decreased, the structural barrier formed by the glycocalyx is lowered and ACE2 expression is upregulated. Hence, endothelial cells might be more susceptible to the virus entry. Significantly, endothelial dysfunction, which is present in inflammation, and cardiovascular disease, diabetes and endothelial ageing is usually related to glycocalyx impairment^40,44^. The presence of the above comorbidities may worsen the prognosis, frequently causing a severe COVID-19 coarse and might often cause of death^5^. Our results indicate that the reduction of endothelial glycocalyx may explain those observations.

Concluding, glycocalyx has the main contribution to the attachment of viral S-protein to ECs. Simultaneously, glycocalyx shields the S-protein interaction with ACE2 whereas its removal exposes ACE2 on the cell surface. In our experiments, we monitored the adhesion of isolated S-proteins at short time scales. Therefore, the results should be benchmarked with studies for active viruses. However, the increased number of reports that SARS-CoV-2 virus penetrates the bloodstream and may directly affect the endothelium make this observation of particular importance.

## Methods

### Cell culture

Primary Human Bronchial Epithelial Cells (HBECs, ATCC) cells were cultured in Airway Epithelial Cell Basal Medium (Cat. No. PCS-300-030, ATCC) supplemented with Bronchial Epithelial Cell Growth Kit (Cat. No. PCS-300-040, ATCC). Primary Human Pulmonary Artery Endothelial Cells (HPAECs, ATCC) were grown in Vascular Cell Basal Medium (Cat. No. PCS-100-030, ATCC), supplemented with Endothelial Cell Growth Kit-VEGF (Cat. No. PCS-100-041, ATCC) The cells were maintained in standard conditions at 37 °C, 5% CO_2_, and 95% humidity.

### Transfection with small interfering RNA

Transfections of HPAECs were performed using 20 nM siRNA targeted against human ACE2 (Life Technologies, s11056) or mock siRNA (Life Technologies; Negative Control #2) using Lipofectamine™ RNAiMAX Transfection Reagent (Life Technologies, s33966) in Opti-MEM I Reduced Serum medium (Life Technologies). The cells were used for the experiments at 48 h after transfection.

### Quantitative RT-PCR

RNA was isolated using RNeasy Mini Kit (Qiagen). Reverse transcription reaction was made using High Capacity cDNA Reverse Transcription Kit (Life Technologies). The procedures were performed according to the manufacturer’s instructions. Quantitative RT-PCR was performed using StepOnePlus real-time PCR system (Applied Biosystems) with SYBR Green PCR Master Mix (Sigma-Aldrich). Sequences of used primers: h*ACE2* F: GGACCCAGGAAATGTTCAGA R: GGCTGCAGAAAGTGACATGA; h*EEF2* F: TGAGCACACTGGCATAGAGGC R: GACATCACCAAGGGTGTGCAG.

### QI-AFM imaging

HBEC and HPAEC AFM imaging was performed using V-shaped gold-coated cantilevers (MLCT, Bruker) with nominal spring constant of 0.03 N/m. All experiments were performed for non-fixed cells in Hanks’ Balanced Salt Solution (H8264, Sigma-Aldrich). Images (128 × 128 pixels) were obtained at scan size of 20 × 20 μm^2^ for HBECs and 40 × 40 μm^2^ for HPAECs. Topographical images were performed using force-distance (FD)–based imaging mode (QI; JPK Instruments) allowing for high-resolution imaging of living cells. In this method, single FD-curve is performed in every pixel point of the image and then translated from the selected trigger force into images of cell topography. The loading force used varied from 0.8 to 1.4 nN and was adjusted to obtain a clear contrast of the cell surface. The obtained images of topography were analyzed using JPK Data Processing Software.

### AFM probe covering with S-proteins

For measurement, we used spherical glass probes, with a radius of 1.25 μm, attached to a flexible cantilever with the spring constant of 0.02 N/m (Novascan). First, each probe was cleaned with ethanol and dH_2_0, then incubated with 10% APTES (Sigma Aldrich) in dH_2_0 for 20 min at room temperature (RT). After this time, probes were rinsed accurately three times with dH_2_0 and were immersed in 2.5% Glutaraldehyde (Sigma Aldrich) for 15 min. Next, the probes were rinsed in dH_2_O and immersed in 50 ng/mL Anti-His-Tag (BioVision) solution (PBS) for 15 min at RT. At the end, the probes were incubated with 50 ng/mL Recombinant Coronavirus Spike Protein (SARS-CoV S1, His-Tag, BioVision) solution for 30 min. Each probe was very gently rinsed and immediately taken for measurements. One S-protein covered probe was used for experiments on a maximum 4 cells (Supplementary Figure 5).

### Force-distance curve measurement

For AFM experiments, HPAECs or HBECs were seeded at a density of 10^4^ cells/mL on a round glass coverslip and then cells were grown for 48 h. Next, the sample with cells was gently mounted into AFM liquid cell (BioCell, JPKInstruments) and measured in Hanks’ Balanced Salt Solution (HBSS, Sigma-Aldrich) supplemented with 4.5 mM glucose at stable temperature 36.2 °C.

All AFM measurements were performed using a NanoWizard 3 NanoScience AFM (JPK Instruments). Measurements were conducted using a force mapping mode. For each cell, a spatial map of force vs distance (FD) curves at a grid of 7 × 7 or 16 × 16 points was measured. The size of the grid corresponds to a square surface with dimensions from 10 μm × 10 μm to 25 μm × 25 μm. The resulting step size of approx. 1.5 μm was chosen to be comparable with the probe diameter. The position of scan areas was controlled by inverted optical microscopy (Olympus). Force-distance curves were measured at a speed of 2 µm/s. The contact time was 1 s, and the maximal applied force was 500 pN (Supplementary Figure 4). Before and after a series of force-distance measurements, calibration of cantilever spring constant was performed by using Nanowizard software.

To evaluate changes of the elastic modulus of HPAEC after incubation with S-protein, AFM measurements were performed with a non-covered spherical polystyrene AFM probe with a radius of 2.2 μm (Novascan) mounted on the triangular cantilever with the spring constant 0.03 N/m.

### Force-distance curve analysis

To determine the cell elastic modulus E and glycocalyx length L, we used a procedure proposed by Sokolov et al.^54^. As described in our recent papers^41^, the Hertz model was fitted to the part of the curve close to the maximal indentation, for which the glycocalyx is assumed to be almost squeezed. According to Alexander-de Gennes theory, the steric polymer brush model was fitted to the data to calculate the length of the glycocalyx layer. The parameters were derived using software written in Matlab environment.

The adhesive parameters were obtained by the analysis of the retract part of force-distance curves by using JPKSPM Data Processing software. Maximal detachment force F^max^ was calculated as the outermost point on the retraction curve with respect to the baseline. The parameter F^max^ corresponds to the force required to detach the probe covered S-protein from the cells surface and reflects the non-specific interaction as well as the specific interaction of S-protein with the cells surface. Rupture events are defined as characteristic unbinding jumps recorded on the FD curve associated with the breakage of specific bonds between membrane receptors and the S-protein.

### Design of AFM experiments

At first, the native cells were measured. To block the specific interactions, a 100 ng/mL anti-ACE2 (BioVision) blocking antibody solution was added to the native cell and incubated for 20 min in AFM liquid cell. After this time, the buffer was replaced, AFM probe coated with S-protein was mounted and the force-distance (FD) curves were immediately measured. To block S-proteins, the AFM probe covered with S-protein and cells were exposed to 50 U low molecular weight heparin (LMWM, Sigma) for 30 min. After this time, FD curves were measured. For reduction of glycocalyx layer, 100 U Heparinase I/III (Sigma) was added to the native cells immersed in HBSS and incubated for 45 min at 36.2 °C in AFM liquid cell. After this time, the buffer was replaced and the AFM measurement started. In the end, the anti-ACE2 was added to block the ACE2 receptors. All experiments were repeated two or three times, as described in Supplementary Table 1.

### Heparan sulfate and ACE2 staining of HPAECs

48 h before the experiment, cells were seeded on coverslip coated with 0.1% gelatin (Sigma). At the experimental day, cells were incubated with 10 U heparinase (Sigma Aldrich) for 3 h. After treatment, cells were washed with PBS and then fixed with 4% paraformaldehyde solution in PBS (ChemCruz) for 10 min in RT. Next, cells were briefly washed 3 times with PBS and blocked for 1 h in RT with a blocking solution containing 5% BSA (Sigma) in PBS. Then cells were incubated O/N with I antibody against HS (1:100, Amsbio cat. clone F58-10E4) and ACE2 (1:100, BioVision) in blocking solution at 4 °C. Next day, cells were washed 3 times with PBS (5 min, RT) and incubated with II antibody conjugated with a fluorophore (1:500) (Life Technologies), subsequently counterstained with DAPI (Sigma Aldrich) in blocking solution for 1 h at RT. After this step, cells were washed 3 times with PBS (5 min, RT), mounted with DAKO Mounting Medium (Dako) to the glass slides and analyzed with a meta laser scanning confocal microscope (LSM-880; Carl Zeiss). The fluorescence intensity data was measured by using ImageJ2x software.

### Glycocalyx visualization

48 h before the experiment, cells were seeded on coverslip coated with 0.1% gelatin (Sigma). After the appropriate time, cells were fixed with paraformaldehyde (3.8%, Sigma) for 10 min. Next, cells were gently rinsed in PBS three times and incubated with blocking peptide solution (Santa Cruz Biotechnology) for 30 min. After this time, they were washed three times in PBS and incubated with wheat germ agglutinin (Wheat Germ Agglutinin, Alexa Fluor™ 555 Conjugate, Invitrogen) for 30 min.

### Actin staining of HPAEC

HPAECs were grown on a coverslip coated with 0.1% gelatin (Sigma) for 48 h. Before staining, the culture medium was removed, the cells were rinsed with PBS and then fixed with 3.6% formaldehyde (Sigma) for 10 min in room temperature. Next, the cells were rinsed three times with buffer and permeabilized with 0.1% Triton X (Invitrogen-ThermoFisher) for 4 min, followed by blocking in PBS containing 1% BSA (Invitrogen-ThermoFisher) for 30 min. After this time, cells were rinsed with PBS and incubated with phalloidin conjugated with Alexa Fluor 488 dye (1:8000, Molecular Probes) for 20 min and counterstained with Hoechst (Sigma Aldrich) for 5 min at RT. Before the measurement, cells were rinsed twice with PBS and mounted with DAKO Mounting Medium (Dako) to the glass slides. Fluorescence images were obtained using a confocal microscope (LSM-880; Carl Zeiss). The fluorescence intensity data was measured by using ImageJ2x software.

### Statistical analysis

The force spectroscopy AFM data and nanoindentation data were presented in the form of histograms. The bar size for maximal adhesion force is 20 pN. The mean values were calculated based on a log-normal distribution fit, and presented as a mean value ± SD. The fluorescence intensity data of HS and ACE2 were presented in the form of box-plot; each point corresponds to the fluorescence intensity of single-cell normalized to the cell surface. For phalloidin, the fluorescence intensity was measured for all image and normalized to the number of cells. The data were presented as a mean value ± SD. Statistical significance was tested using single-way ANOVA (P < 0.05) followed by Tukey’s mean comparison tests. Since the AFM data were log-normally distributed, we performed data transformation using a natural logarithm, in order to use the ANOVA statistical test. In the manuscript, all data was shown as non-transformed raw data. All statistical analysis and graphs are prepared in Origin.

## Supplementary Information

Supplementary Information.

## Data Availability

The source data underlying Figs. 3a,g, 4b,e,h,f,i, 5a,b,e,f and Supplementary Figures 1 and 2 are provided as a Source Data file. All other relevant data are available from the corresponding authors upon reasonable request.
