# Supplementary material for: Endothelial glycocalyx shields the interaction of SARS-CoV-2 spike protein with ACE2 receptors
Source: Sci Rep. 2021 Jun 9;11:12157. doi: 10.1038/s41598-021-91231-1 (PMC8190434; doi:10.1038/s41598-021-91231-1)
Supplement: Supplementary file 1 — Supplementary Information. [file 41598_2021_91231_MOESM1_ESM.pdf]

Supplementary information

Endothelial glycocalyx shields the interaction of SARS-CoV-2 spike protein with ACE2 receptors

Marta Targosz-Korecka[1*],Agata Kubisiak[1], Damian Kloska[2], Aleksandra Kopacz[2], Anna Grochot-Przeczek[2], Marek Szymonski[1]

[1]Department of Physics of Nanostructures and Nanotechnology, Faculty of Physics, Astronomy and Applied Computer Science, Jagiellonian University, Krakow, Poland

[2]Department of Medical Biotechnology, Faculty of Biochemistry, Biophysics and Biotechnology, Jagiellonian University, Krakow, Poland

Experiment details

Supplementary Table 1. Summary of studied systems. N – number of cells. n – number of experiments with different AFM probes on different samples. k – total number of measured force-distance curves. Anti-ACE2 – ACE2 blocking antibody, Hase – heparinase.

**Cell type**	**System**	**Figure**	**N**	**n**	**k**
**HBEC**	native	2A	9	3	1142
	anti-ACE2	2A	5	2	393
	heparine	2A	5	2	256
**HPAEC**	native	2E	8	3	980
	anti-ACE2	2E	5	2	904
	heparine	2E	3	2	511
	Hase	3D-G	5	2	847
	Hase + anti-ACE2	3E-H	3	2	194
	S-protein	4A	6	2	314
	Hase + S-protein	4D	6	2	224

Supplementary Table 2. Summary of mean values calculate for all studied systems. This values were calculated based on histograms presented in Suplementary Figure S2 and Suplementary Figure S3

	**Native**	**+Ab-ACE2**	**+Heparin**	**Hase**	**+Hase +Ab-ACE2**
Mean max. adhesion force F^{max} [pN]					
HPAEC	195±2	190±4	108±6	126±7	70±9
HBEC	286±12	111±10	121±6	-	-
Mean number of adhesion steps (No. Steps)					
HPAEC	5±1	4±1	2±1	7±1	2±1
HBEC	15±1	6±1	7±1**	-	-
Work of detachment [fJ]					
HPAEC	0.15±0.03	0.29±0.06	0.05±0.01	0.13±0.05	0.08±0.03
HBEC	0.1±0.01	0.06±0.01	0.08±0.01	-	-
Mean Elastic modulus [Pa]					
HPAEC	1622±5	2268±15	1258±14	693±7	3195±18
HBEC	1273±8	1899±12	1315±6	-	-
Mean glycocalyx length [nm]					
HPAEC	333±3	269±4	337±6	177±8	123±4
HBEC	854±14	782±14	790±10	-	-

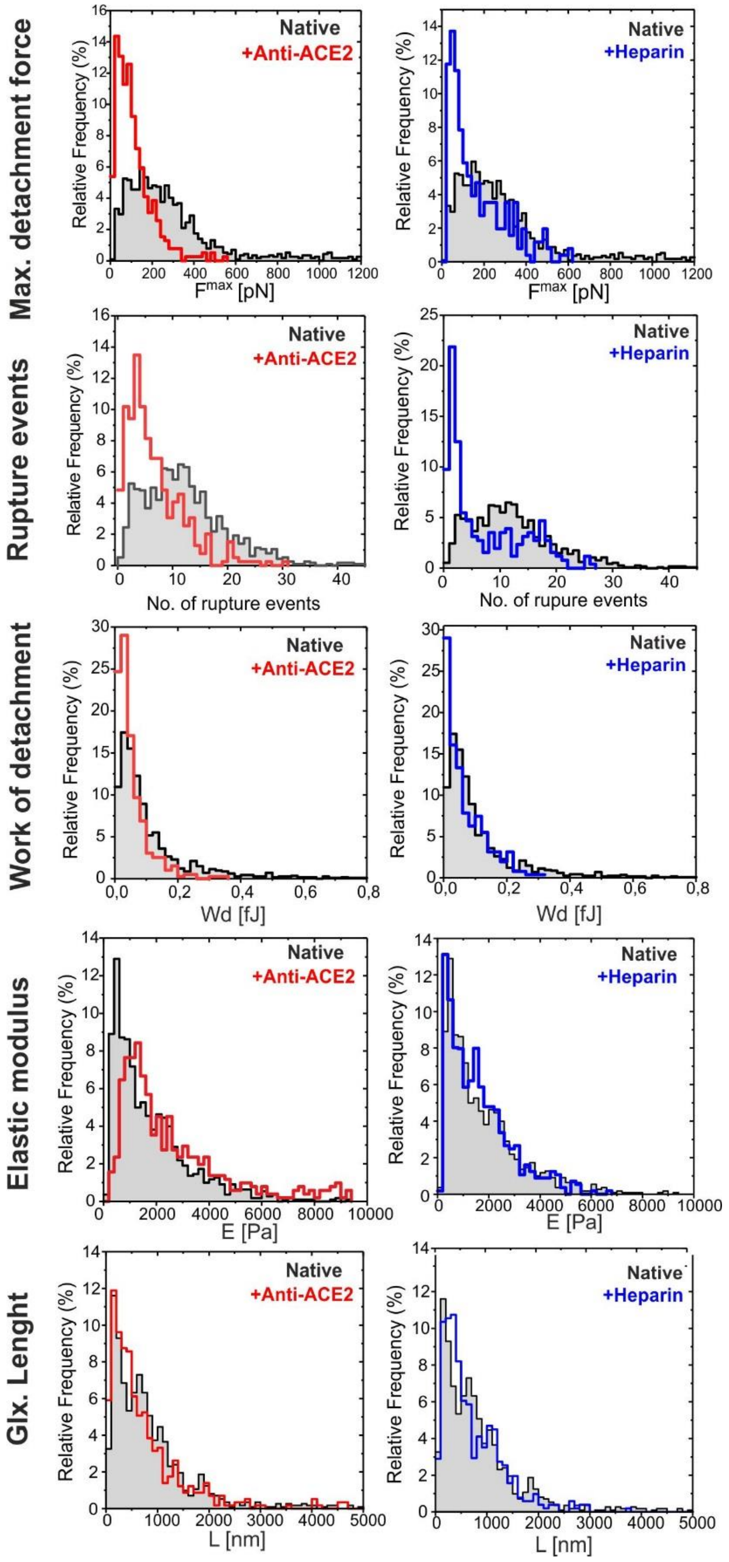

Suplementary Figure 1. Summary of all histograms for HBECs. Note that for HBECs, the determined glycocalyx length may have a large systematical error due to the presence of cilia on HBEC surface. A single brush model used in our analysis method is unable to take this effect into account [40]. Anti-ACE2 – ACE2 blocking antibody, glx – glycocalyx, Wd - work of detachment (area under retract part of FD). Figure created with OriginPro 2021.

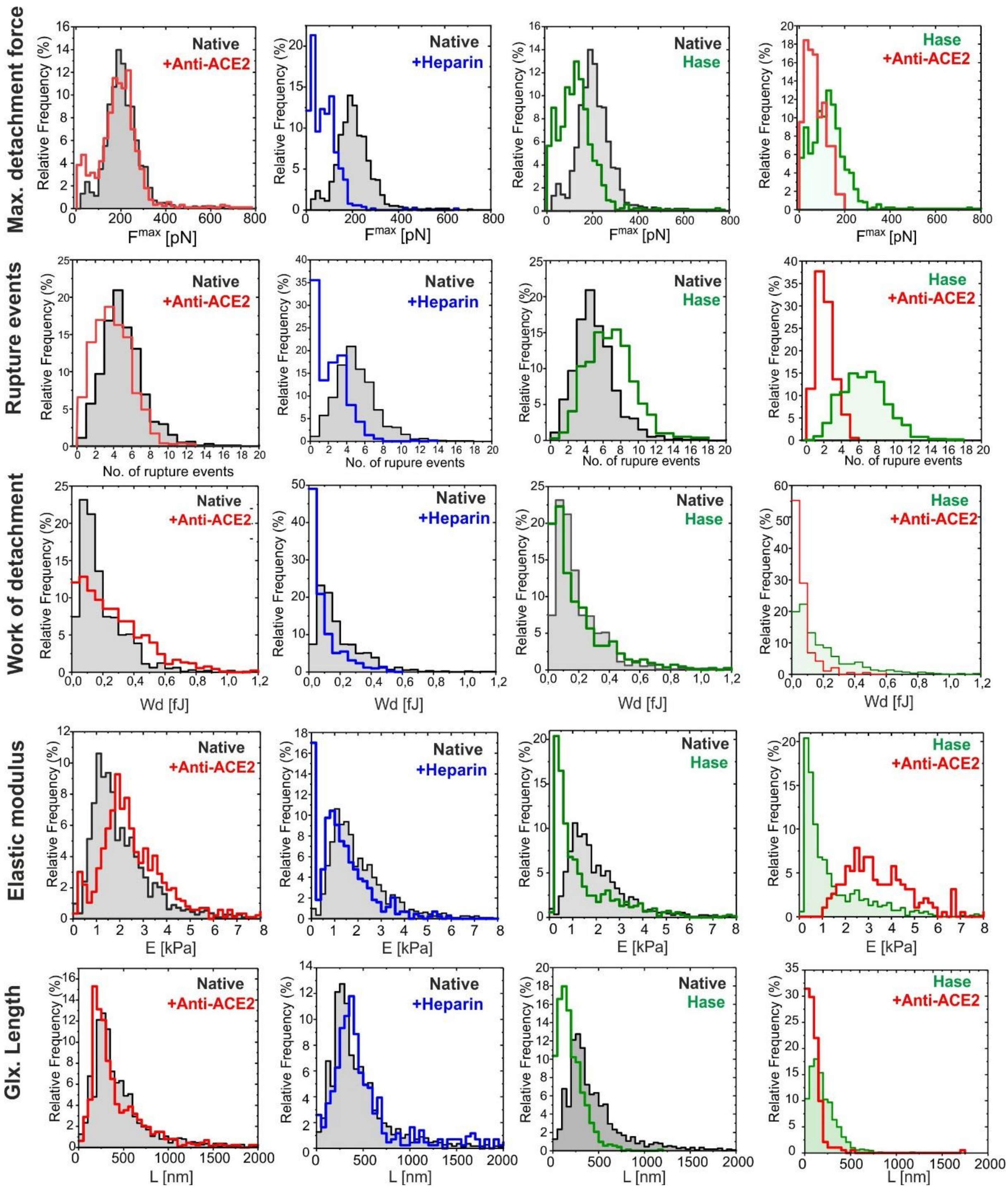

Supplementary Figure 2. Summary of all histograms for HPAECs. Glx – glycocalyx, anti-ACE2 – ACE2 blocking antibody, Hase – heparinase, Wd - work of detachment (area under retract part of FD). Figure created with OriginPro 2021.

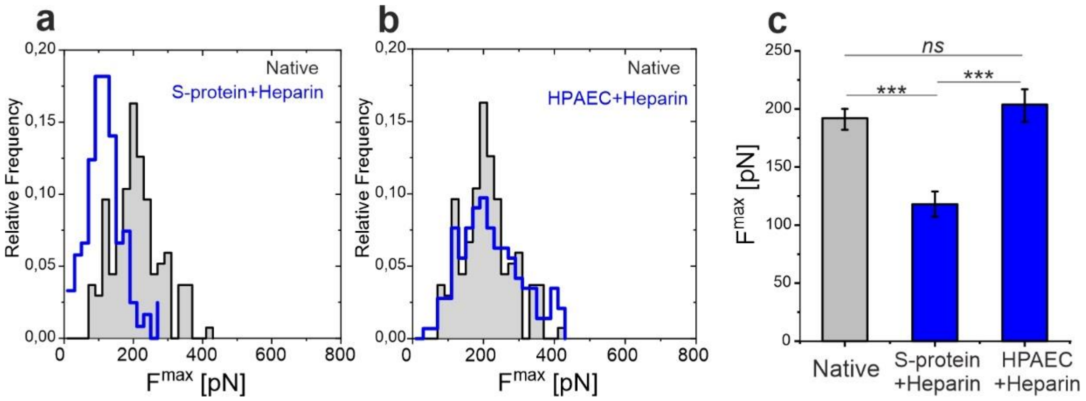

Supplementary Figure 3. Selective incubation of HPAECs and S-protein on the AFM probe with heparin. a Histograms of maximal detachment force between native HPAEC and S-protein. Only the probe was incubated with Heparine. **b** Only the cells were incubated with heparine. **c** Comparison of mean values. Statistics: one-way ANOVA followed by Tukey's post-hoc test. (*) $p<0.05$, (***) $p<0.001$, (ns) non-significant, N=8, n=2. Figure created with OriginPro 2021.

Determination of the experimental conditions

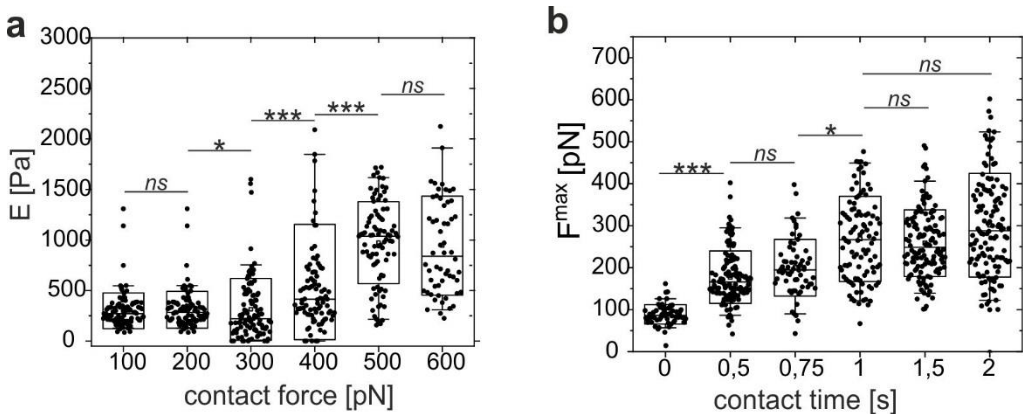

Supplementary Figure 4. Determination of the force spectroscopy experimental conditions. a Determination of experimental contact force value. Elastic modulus determined from the approach part of the curve vs the contact force. Visible stabilization for the contact force of 500 pN. **b** Determination of experimental contact time value. Measured maximal detachment force (F^{max}) vs contact time for native cells. After an initial increase, F^{max} was stabilized for the contact time higher than 1s. Statistics: one-way ANOVA followed by Tukey's post-hoc test. (*) $p<0.05$, (***) $p<0.001$, (ns) non-significant. Figure created with OriginPro 2021.

During a force curve measurement, the bonds between S-proteins and endothelial cell surface are formed under the influence of an external mechanical force. Therefore, it is essential to control two crucial parameters: the contact force and the contact time. Contact force is defined as the maximum force that is sensed by the probe during the approach phase. Supplementary Fig. S5a evaluates the derived value of the apparent elastic cell modulus for different applied contact force values. For further experiments, a value of 500 pN was set, which corresponds to a plateau in the observed data. Simultaneously, the derived value of apparent elastic the modulus is at the level of 1 kPa what denotes

that the probes almost entirely squeezes the glycocalyx layer and senses the cell body. The contact time determines the adhesion probability and the number of formed bonds between S-protein and the cell surface. This parameter was set to 1s. For longer contact times, the adhesion force did not change significantly, as shown in Supplementary Fig S5b

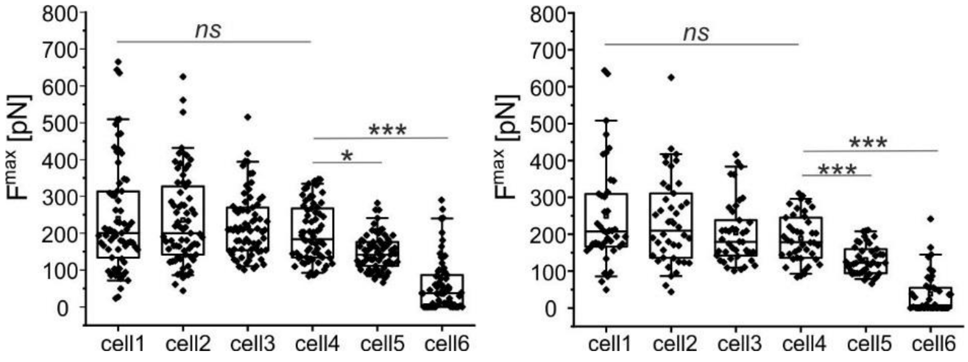

Supplementary Figure 5. AFM probe coverage stability test. Two independent measurements of maximum detachment force for multiple cells performed with S-protein covered AFM probe. After 4 cells the data significantly differ what suggests the probe contamination or S-proteins removal. Based on these observations, one probe has been used for maximum 4 cells measurements. Statistics: one-way ANOVA followed by Tukey's post-hoc test. (*) p<0.05, (***) p<0.001, (ns) non-significant. Figure created with OriginPro 2021.
